# Editorial: Interplay between NO Signaling, ROS, and the Antioxidant System in Plants

**DOI:** 10.3389/fpls.2016.01731

**Published:** 2016-11-16

**Authors:** Jeremy Astier, Gary Loake, Violeta Velikova, Frank Gaupels

**Affiliations:** ^1^Department of Environmental Science, Helmholtz Zentrum München, Institute of Biochemical Plant PathologyNeuherberg, Germany; ^2^Institute of Molecular Plant Sciences, School of Biological Sciences, University of EdinburghEdinburgh, UK; ^3^Photosynthesis - Activity and Regulation, Institute of Plant Physiology and Genetics, Bulgarian Academy of SciencesSofia, Bulgaria

**Keywords:** reactive oxygen species, nitric oxide, antioxidant system, plant defense, biotic and abiotic stress, plant development

Over the last decades, reactive oxygen and nitrogen species (ROS and RNS), and particularly nitric oxide (NO), have been linked to a wide variety of physiological processes in plants, ranging from the control of developmental processes to the regulation of plant responses against biotic or abiotic stress. In addition to the regulation of ROS and RNS biosynthesis, the exquisite modulation of their activities is also under the control of a complex network of regulatory enzymes and compounds constituting the antioxidant system. The Research Topic on the *Interplay between NO signaling, ROS and the antioxidant system in plants* aims to provide an up-to-date view of this increasingly important area through both original articles and detailed reviews.

NO and ROS signaling pathways are key regulators of plant development. In this context, Ma et al. studied the role of NO, ROS, and the antioxidant system on the germination of barley seeds. They found that the turnover of NO via the action of phytoglobin and S-nitrosoglutathione reductase and also its interaction with ROS contributed to the alleviation of seed dormancy. The impact of oxidative stress on plant development is also outlined in the mini-review presented by Frank and Ernst where they summarize the current knowledge about the effect of the air pollutants NO_2_ and ozone on pollen allergenicity. The authors report that both NO_2_ and ozone result in increased pollen allergenicity, in a dose-dependent and species-specific manner. Further, corroborating the pivotal role of ROS in developmental processes, Jiménez-Quesada et al. summarize in an extensive review the importance of NADPH oxidase activity on sexual plant reproduction mechanisms.

The interplay between NO and ROS in the early development of the symbiotic interaction between plants and rhizobium partners is also reviewed in this Topic by Damiani et al. They describe how the spatiotemporal accumulation of these compounds is tightly regulated for the successful establishment of the symbiosis, both in the plant host and the bacteria symbiont.

The role of NO, ROS, and the antioxidant system during plant/pathogen interactions is also addressed in this Topic. Thalineau et al. studied their role the infection of *Medicago truncatula* by *Aphanomyces euteiches*. They demonstrate that NO homeostasis impacts nitrate reductase activity and therefore N nutrition. They propose a link between NO/ROS homeostasis, N nutrition and plant immunity. Gaupels et al. explored the long distance signaling induced in the extrafascicular phloem of pumpkins during systemic wound responses. They report that a decrease in the antioxidant system capacity correlates with an increase in NO/ROS content. Sivakumaran et al. reveal an antagonistic role of abscisic acid on NO/ROS generation in tomato following challenge with *Botrytis cinerea*. They propose this mechanism might be a plant defense suppressing strategy exhibited by the pathogen enabling the development of a compatible interaction. The significance of ROS and NO during biotic interactions from the pathogen perspective is also developed in this Topic. Arasimowicz-Jelonek and Floryszak-Wieczorek review this question focusing on the specific role of NO in pathogenic fungi and oomycetes. In an original work, Yin et al. show that both NO and ROS are crucial players in the germination of urediniospores of *Puccinia striiformis* f. sp. *tritici*, the agent responsible for stripe rust in wheat.

Another important feature of this Topic focuses on the plant responses to abiotic stresses. Farnese et al. provide a comprehensive overview of NO, ROS, and the antioxidant system in plant adaptive responses to abiotic stress, emphasizing their crosstalk and specificities depending on the given stress. Fatma et al. underline the effect of NO and sulfur on photosynthetic performance of mustard plants exposed to salt stress, demonstrating that the combined application of NO and S provides the best protection of photosynthetic machinery. In an original article, Lytvyn et al. report the link between inositol and NO signaling in the development of oxidative stress following ultraviolet-B irradiance in *Arabidopsis thaliana*. Also, Wu et al. describe the relation between NO and heme oxidase 1 in germinating rice aleurone layer subjected to drought stress. They describe how the mutual induction of these two factors affects gibberellin-induced programmed cell death following germination. In addition to these original data, Molassiotis et al. discuss in a mini-review the mechanism associated with NO and H_2_O_2_ priming of citrus defense responses prior to abiotic stress, whereas Gupta and Igamberdiev review the crucial role of mitochondria in modulating RNS and ROS signaling to promote plant survival on exposure to hypoxia or anoxia.

The better understanding of the NO- and ROS-dependent biochemical mechanisms themselves is another aspect developed in this topic. Krasuska et al. analyzed the mode of action of the tomato root growth inhibitor canavanine (CAN), a non-proteogenic amino acid and a structural analog of arginine, used as a mammalian nitric oxide synthase inhibitor. They conclude that, despite decreasing NO levels, the rapid response of plants to CAN exposure probably results from an increase in carbonylated proteins following ROS production. The post-translational modification of proteins is also the main subject reviewed by Begara-Morales et al. These authors detail our present knowledge regarding the NO modification of enzymes integral to the plant antioxidant system, with a particular focus on the components of the ascorbate-glutathione cycle. Further, Romero-Puertas and Sandalio discuss how NO might regulate its own levels and that of its ROS partners. They discuss the important factors integral to the fine tuning existing between NO and ROS signaling. Finally, Gross and Durner reviewed recent research data pertaining to the NO-3′,5′-cyclic guanosine monophosphate (cGMP) dependent pathway in plants. While NO mediated cGMP signaling is well described in mammals, this system is not well defined in plants. These authors discuss the current situation concerning the identification of enzymes involved in such a pathway in higher plants.

The wide variety of the work reported here testifies to the importance of NO, ROS, and the antioxidant system to plant physiology, and highlights the complexity of their interplay. We hope that this Topic will provide an update of this important and rapidly expanding area and also offer a primer to drive future exciting lines of research enquiry.

## Author contributions

All authors listed, have made substantial, direct and intellectual contribution to the work, and approved it for publication.

### Conflict of interest statement

The authors declare that the research was conducted in the absence of any commercial or financial relationships that could be construed as a potential conflict of interest.

